# Chinese environmentally extended input-output database for 2017 and 2018

**DOI:** 10.1038/s41597-021-01035-1

**Published:** 2021-09-30

**Authors:** Xi Tian, Yiwei Liu, Ming Xu, Sai Liang, Yaobin Liu

**Affiliations:** 1 Research Center for Central China Economic and Social Development, Nanchang University, Nanchang, 330031 China; 2 Jiangxi Ecological Civilization Research Institute, Nanchang University, Nanchang, 330031 China; 3 School of Economics and Management, Nanchang University, Nanchang, 330031 China; 4 School for Environment and Sustainability, University of Michigan, Ann Arbor, Michigan 48109-1041 United States; 5 Department of Civil and Environmental Engineering, University of Michigan, Ann Arbor, Michigan 48109-2125 United States; 6 Key Laboratory for City Cluster Environmental Safety and Green Development of the Ministry of Education, Institute of Environmental and Ecological Engineering, Guangdong University of Technology, Guangzhou, Guangdong 510006 China

**Keywords:** Environmental impact, Environmental impact

## Abstract

Environmental footprint analyses for China have gained sustained attention in the literature, which rely on quality EEIO databases based on benchmark input-output (IO) tables. The Chinese environmentally extended input-output (CEEIO) database series provide publically available EEIO databases for China for 1992, 1997, 2002, 2007, and 2012 with consistent and transparent data sources and database structure. Based on the latest benchmark IO tables for China for 2017 and 2018, here we develop the corresponding 2017 and 2018 CEEIO databases following the same method used to develop previous CEEIO databases. The 2017 and 2018 CEEIO databases cover 44 and 28 types of environmental pressures, respectively, and consider multiple sector classifications including ones consistent with previous CEEIO databases and ones following the 2017 China’s national economy industry classification standard. A notable improvement in the 2017 and 2018 CEEIO databases is the comprehensive inclusion of CO_2_ emissions from additional industrial processes. This work provides a consistent update of the CEEIO database and enables a wide range of timely environmental footprint analyses related to China.

## Abstract (summary)

**Table:** 

Measurement(s)	environmental pressure
Technology Type(s)	digital curation
Sample Characteristic - Location	China

Machine-accessible metadata file describing the reported data: 10.6084/m9.figshare.15656622

## Background & Summary

Environmental footprints account for the amounts of environmental pressures generated by all sectors of an economy as the result of final demand including consumption, capital formation, and exports. Environmental footprint analysis helps develop consumption-side strategies and policies to mitigate economy-wide environmental pressures through the whole supply chain. The environmentally extended input-output (EEIO) model is the method used widely to evaluate environmental footprints. The core of the EEIO model is an EEIO database, which consists of an economic input-output (EIO) table and an environmental satellite account.

As the world’s second largest economy, leading consumer of various resources and energy, and dominating producers of emissions and waste, China has been one of the foci in environmental footprint analyses. China-specific EEIO models have been developed and applied to study a variety of environmental footprints in China such as carbon footprint^1–4^, water footprint^5–7^, energy footprint^8,9^, material footprint^10,11^, and atmospheric Hg footprint^12–14^. While the data sources used to construct EEIO databases in these studies are largely the same, the developed EEIO databases are rarely made publically available. To the best of our knowledge, the Chinese Environmentally Extended Input-Output (CEEIO) database that we developed is the only publically available EEIO database for China at the national level (www.ceeio.com)^15^. Note that there are open access multi-regional EEIO databases available for China at the provincial level^16,17^.

The latest CEEIO database (version 2.0) includes EEIO models for China in 1992, 1997, 2002, 2007, and 2012 covering 256 types of resources and 31 types of pollutants. The database also comes with multiple sector classifications including the classifications from the corresponding benchmark EIO tables which vary across years, and two consistent classifications across years with 45 and 91 sectors, respectively. The economic transactions in the CEEIO database are also characterized in both Chinese yuan and US dollar.

The most recent benchmark EIO tables for China are for 2017 and 2018. Yet China-specific EEIO models for 2017 and 2018 are not publicly available. In this work, we develop 2017 and 2018 EEIO models for China as part of the CEEIO database and make it publicly available at www.ceeio.com. To develop the 2017 and 2018 CEEIO databases, we collect data on energy consumption, industrial product production, and pollutant releases in China in 2017 and 2018 from relevant government statistics. The emission factors of fuel combustion and industrial processes are from the latest research in the literature.

There are three major contribution of Our 2017 and 2018 CEEIO databases. First, to the best of our knowledge, the CEEIO database covers the most categories of environmental pressures and industrial processes compared to other similar data. Compared to the previous versions, we newly added 12 types of hazardous trace elements which have significant public health impacts. Utilizing the CEEIO databases, one can easily calculate the footprints of those environmental pressures and identify the key sectors responsible for those emissions. Second, we quantified the uncertainties of the emission inventories. Applying Monte Carlo simulation, we have quantified the integrated uncertainties in the emissions of 13 types of environmental pressures based on data from relevant research. Third, multiple sector classifications are offered. We provide CEEIO databases of multiple sectoral classifications for flexible use. To be consistent with the previous versions, we have merged the 149 sectors in the benchmark input-output tables into 45 and 91 sectors. We also present the CEEIO database in 49- and 96-sector classifications to conform with the new national economy industry classification standard used since 2017.

## Methods

### Scope

We consider four broad categories, 44 types of environmental pressures generated by domestic sectors and households in China for the 2017 CEEIO database based on the available data: (1) freshwater consumption; (2) 23 types of atmospheric pollutants including carbon dioxide (CO_2_), methane (CH_4_), nitrous oxide (N_2_O), nitrogen oxides (NO_x_), dust and soot, sulfur dioxide (SO_2_), hazardous trace elements (HTEs, including Hg, As, Se, Pb, Cd, Cr, Ni, Sb, Mn, Co, Cu, and Zn), particulate matter with an aerodynamic diameter of 2.5 mm or less (PM_2.5_), particulate matter with an aerodynamic diameter of 10 mm or less (PM_10_), carbon monoxide (CO), Volatile Organic Compounds (VOCs), ammonia (NH_3_); (3) 13 types of water pollutants including chemical oxygen demand, ammonia nitrogen compounds, phosphorus, petroleum pollutants, volatile phenols, cyanide, aquatic Hg, aquatic Cd, aquatic Cr, aquatic Pb, aquatic As, aquatic Cu, and aquatic Zn; and (4) 7 types of solid waste including industrial solid waste, plastic film, crop straws, animal manure, sludge, medical waste, and household waste. The 2018 CEEIO database covers 28 types of environmental pressures among these 44 based on the available data: (1) the fuel combustion from sectors^18^, (2) the output of industrial products^19^, (3) the yield of crop, (4) the amount of livestock and poultry, (5) the freshwater consumption for agriculture, industry, and domestic use^20^, and (6) the usage amount of plastic film^21^.

### Data sources and estimation for environmental pressures

Here we show how the 2017 CEEIO database is developed in detail, while the 2018 database is developed in similar way. Compared with the previous CEEIO database, the major improvements include (1) updated emission factors of environmental pressures including greenhouse gas, nitrogen oxides, atmospheric Hg, atmospheric As, and atmospheric Se in the combustion process of coal, natural gas, and petroleum products, (2) covered additional industrial process (e.g., the production of cement, synthetic ammonia, and coke) when accounting for the emissions of nitrogen oxides, (3) added 14 types of environmental pressures including Atmospheric Pb, Atmospheric Cd, Atmospheric Cr, Atmospheric Ni, Atmospheric Sb, Atmospheric Mn, Atmospheric Co, Atmospheric Cu, Atmospheric Zn, PM_2.5_, PM_10_, CO, VOCs, NH_3_, and (4) adjusted the sector classification according to the new national economy industry classification standard adopted since 2017.

**Fig. 1:**
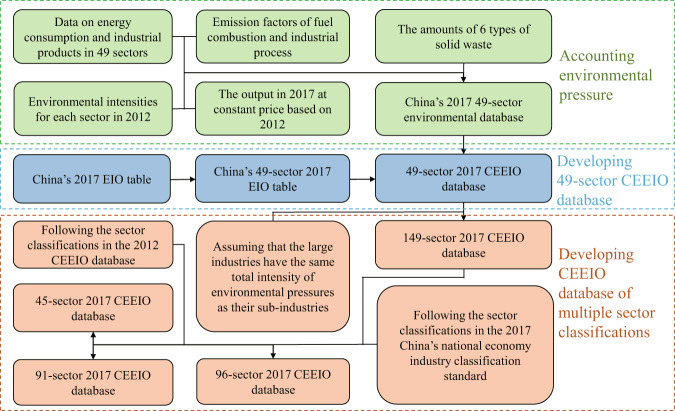
Process of developing the 2017 CEEIO database. The part in green shows the first step of constructing the 2017 CEEIO database where we account the sectoral emission of 44 types of environmental pressure. The blue area shows how we develop the 49-sector 2017 CEEIO database. The orange part shows the construction process of the CEEIO database of different sector classifications.

Figure 1 shows the process of developing the 2017 CEEIO database which also applies to the 2018 one. First, we establish China’s 2017 49-sector environmental account based on the available data. Second, according to the 2017 China’s National Economy Industry Classification Standard, we merge the 149 sectors in China’s 2017 benchmark EIO table and corresponding economic data to 49 sectors to establish the 49-sector CEEIO database. Third, assuming the environmental intensity of an aggregate sector is the same as that of any sub-sector, we develop the CEEIO database with 149 sectors consistent with the 2017 benchmark EIO table. Fourth, we merge the 149 sectors and corresponding environmental pressure data to 45 and 91 sectors, respectively, to be consistent with previous CEEIO databases. Lastly, according to the new sector classifications in China’s 2017 National Economy Industry Classification Standard, we extend the 91-sector classification to develop the 96-sector CEEIO database.

In the 2017 CEEIO database, we include the emissions of 19 types of air pollutants (CO_2_, CH_4_, N_2_O, NO_x_, CO, HTEs, VOCs, NH_3_) from both the combustion of 26 types of fuel sources and industrial processes. Specifically, the 26 types of fuel sources include raw coal, washed coal, other washed coal, briquettes, crude petroleum, natural gas, coke, other coking products, gasoline, kerosene, diesel oil, fuel oil, naphtha, lubricating oil, paraffin oil, solvent oil, bitumen, petroleum coke, other petroleum products, liquefied petroleum gas, coke oven gas, blast furnace gas, converter gas, other gas, liquefied natural gas (LNG), and refinery dry gas. Industrial processes considered in this study are processes producing crude petroleum, natural gas, refined edible vegetable oil, paper, coke, sulfuric acid, caustic soda, soda ash, ethylene, ammonia, chemical fertilizer, primary plastic, synthetic rubber, cement clinker, lime, glass, pig iron, crude steel, rolled steel, copper, electrolyzed aluminum, and alumina oxide. The amounts of emissions of the 19 air pollutants are calculated using Eq. 1:

1 $$A{P}_{i}^{j}=\mathop{\sum }\limits_{k=1}^{26}{\varepsilon }_{k}^{ij}{c}_{k}^{i}+\mathop{\sum }\limits_{k=1}^{n}{\gamma }_{k}^{j}{p}_{k}^{i}$$

where $A{P}_{i}^{j}$ is the discharge of the *j*^*th*^ air pollutant generated by the sector *i*, ${\varepsilon }_{k}^{ij}$ is the emission factor of the *j*^*th*^ air pollutant from the combustion of the *k*^*th*^ type of fuel source in sector *i*, ${c}_{k}^{i}$ is the total amount of the *k*^*th*^ type of fuel consumed by the sector *i*, ${\gamma }_{k}^{j}$ is the emission factor of the *j*^*th*^ air pollutant for the industrial process of producing product *k*, ${p}_{k}^{i}$ is the total amount of *k* produced by the sector *i*, and n is the number of products from sector *i* which varies across sectors. Data for ${\varepsilon }_{k}^{ij}$ and ${\gamma }_{k}^{j}$ are from the Intergovernmental Panel on Climate Change (IPCC)^22–24^, the Ministry of Ecology and Environment of the People’s Republic of China (MEEPRC)^25,26^, and previous studies^27–33^. Besides, the sector of *Electricity & Heat Production and Supply* also consumes fuel sources in their energy conversion process. Therefore, we consider the air pollutants generated from the intermediate energy conversion process accordingly. Moreover, we update the NOx emissions of each sector based on the China’s national NOx emissions inventory in 2017 from the Communique of China’s Second National Survey of Pollution Sources (CCSNSPS)^34^.

The amounts of PM_2.5_ and PM_10_ emissions are calculated using Eq. 2:

2 $$A{P}_{i}^{j}=\mathop{\sum }\limits_{k=1}^{26}{\varepsilon }_{k}^{ij}{c}_{k}^{i}\left(1-{r}_{k}^{ij}\right)+\mathop{\sum }\limits_{k=1}^{n}{\gamma }_{k}^{j}{p}_{k}^{i}\left(1-{s}_{k}^{j}\right)$$

where ${r}_{k}^{ij}$ is the average removal rate of the *j*^*th*^ air pollutant (PM_2.5_ or PM_10_) from the combustion of the *k*^*th*^ fuel source in sector *i*, and ${s}_{k}^{j}$ is the average removal rate of the *j*^*th*^ air pollutant in the industrial process of producing product *k*. Data for ${\varepsilon }_{k}^{ij}$, ${\gamma }_{k}^{j}$, ${r}_{k}^{ij}$, and ${s}_{k}^{j}$ are from the MEEPRC^35,36^ and Bai *et al*.^31^. Similarly, we also consider the PM_2.5_ and PM_10_ emissions generated from the intermediate energy conversion process.

The latest data on emissions of SO_2_, soot and dust, 13 water pollutants, and industrial solid waste are for 2015. Therefore, we use the price index of each sector from 2012 to 2017 and calculate the output in 2017 at constant price based on 2012. Then, we account the amount of these 16 environmental pressures from each sector in 2017 by using the corresponding emission intensities for each sector in 2012. Finally, additional adjustments are made based on the total emissions of each pollutant in 2017 from the National Bureau of Statistics of China (NBSC)^37^ and CCSNSPS^34^. Specifically,

3 $$t{p}_{i}^{j}={e}_{i}^{j}\cdot {x}_{i}\cdot TO{P}^{j}/\mathop{\sum }\limits_{i=1}^{51}{e}_{i}^{j}\cdot {x}_{i}$$

where $t{p}_{i}^{j}$ is the amount of the *j*^*th*^ environmental pressure from sector *i* after correction, ${e}_{i}^{j}$ is the total intensity of the *j*^*th*^ environmental pressure in sector *i*, *x*_*i*_ is the gross output of sector *i* at constant price based on 2012, and *TOP*^*j*^ is the total emission of the *j*^*th*^ environmental pressure in 2017.

The NBSC provides the total amount of freshwater consumed for agriculture, industry, and other use (including construction, tertiary, and households) in 2017 and 2018^20,37^. We estimate the freshwater consumption of *Crop Cultivation*, *Forestry*, *Livestock and Livestock Products*, *Fishery*, and *Technical Services for Agriculture* using Eq. 4:

4 $${w}_{i}={W}^{A}\cdot {z}_{w}^{i}/\mathop{\sum }\limits_{j=1}^{5}{z}_{w}^{j}$$

where *w*_*i*_ is the amount of freshwater consumed by sector *i*, ${z}_{w}^{i}$ is the intermediate input from the sector of *Water Production and Supply* to sector *i*, and *W*^*A*^ is the total freshwater consumption of the agriculture sector. The freshwater consumption of mining, manufacturing, and service sectors as well as urban and rural households are obtained by multiplying the output at constant price based on 2012 by the freshwater consumption by unitary output of each sector from the 2012 CEEIO database. The result is scaled to match the aggregate freshwater consumption for these sectors in 2017 and 2018, respectively.

The CCSNSPS provides the amounts of sludge and medical wastes in 2017^25^. The emissions of the two solid wastes in 2018 are estimated by multiplying the total intensity with the output at constant price based on 2017 of *Water Production and Supply* and *Health Services*. We estimate the rural household waste by multiplying the rural population with the generation factor (0.86 kg/capital*day)^38^ of household waste in rural areas in China, while the NBSC provides the amount of urban household waste^20,37^. We use Eqs. 5, 6, and 7 to estimate the amounts of plastic film, crop straws, and animal manure respectively:

5 $$pf=uwr$$

6 $$cs=\mathop{\sum }\limits_{i=1}^{20}{c}_{i}{f}_{i}{d}_{i}$$

7 $$am=\mathop{\sum }\limits_{i=1}^{6}{n}_{i}{m}_{i}{l}_{i}$$

In Eq. 5, *pf*, *u*, *w*, and *r* represent the amount of plastic film waste, the amount of plastic film used in farmlands^34^, scrap rate (0.58), and emission rate (0.20) of plastic film, respectively^39^. In Eq. 6, *cs*, *c*_*i*_, *f*_*i*_, and *d*_*i*_ represent the amount of crop straws, the yield of crop *i*, the generation rate, and emission rate of crop straws of crop *i*, respectively^40,41^. In Eq. 7, *am* is the amount of manure; *n*_*i*_ is the number of livestock and poultry stored at the end of the year (the breeding cycle of pigs is generally 199 days according to the NBSC, so the number of pigs raised is calculated according to the amount of slaughters fattened hogs)^37^; and *m*_*i*_ and *l*_*i*_ respectively represent the generation rate and emission rate of manure of animal *i*.

### Sector classification

The conversions of difference sector classifications follow Eq. 8 to Eq. 12:

8 $$I{I}_{{\rm{target}}}={M}_{149D-{\rm{target}}}I{I}_{149D}{\left({M}_{149D-{\rm{target}}}\right)}^{T}$$

9 $${X}_{{\rm{target}}}={M}_{149D-{\rm{target}}}{X}_{149D}$$

10 $${Y}_{{\rm{target}}}={M}_{149D-{\rm{target}}}{Y}_{149D}$$

11 $${V}_{{\rm{target}}}={V}_{149D}{\left({M}_{149D-{\rm{target}}}\right)}^{T}$$

12 $${E}_{{\rm{target}}}={E}_{149D}{\left({M}_{149D-{\rm{target}}}\right)}^{T}$$

Notations *II*_target_, *X*_target_, *Y*_target_, *V*_target_, and *E*_target_ represent the intermediate input matrix, total output vector, final demand vector, value added matrix, and environmental pressure matrix in the CEEIO database of target sector classifications, respectively, whereas *II*_149*D*_, *X*_149*D*_, *Y*_149*D*_, *V*_149*D*_, and *E*_149*D*_ represent those in the 149-sector 2017 CEEIO database. The binary matrix *M*_149*D*–target_ shows the inclusive relationships between the 149 sectors and the sectors in the target classification. The binary matrix *M*_149*D*–49*D*_, *M*_149*D*–96*D*_, *M*_149*D*–45*D*_, and *M*_149*D*–91*D*_ are showed in the figshare file 1, figshare file 2, figshare file 3, and figshare file 4, respectively^42^.

Supplementary Table 1 shows the differences in sector classification between the 139-sector 2012 CEEIO database and the 149-sector 2017 CEEIO database. The major difference in sector classification between the 45-sector 2012 CEEIO database and the 49-sector 2017 CEEIO database is that the sector *Technical Services for Agriculture* in 2017 consistent with the 2017 China’s national economy industry classification standard (Supplementary Table 2) is merged into *Other Services* in the 45-sector 2012 CEEIO database. The differences in sector classification between the 91-sector 2012 CEEIO database and the 96-sector 2017 CEEIO database are shown in Supplementary Table 3.

### Energy consumption data

Data on the consumption of 26 types of fuel sources of each sector are obtained from China Energy Statistical Yearbook 2018–2019^18,43^. There are 41 industrial sub-sectors of end-use energy consumption and 8 sectors in the national energy balance sheet. We divide the Chinese economy into 51 sectors, as shown in figshare file 5^42^. Data on the output of 22 industrial products are from the China Industrial Statistical Yearbook 2018–2019^19,44^, including (1) crude petroleum, natural gas belong to the sector of *Crude Petroleum and Natural Gas*; (2) refined edible vegetable oil belongs to the sector of *Food Processing*; (3) machine-made paper and paperboard belong to the sector of *Paper and Paper Products* (4) coke belongs to the sector of *Processing of Petroleum, Coking, and Processing of Nuclear Fuel*; (5) sulfuric acid, caustic soda, soda ash, ethylene, synthetic ammonia, and chemical fertilizer belong to the sector of *Raw Chemical Materials and Chemical Products*; (6) primary plastic and synthetic rubber belong to the sector of *Rubber and Plastic Products*; (7) cement clinker, lime, and glass belong to the sector of *Nonmetallic Mineral Products*; (8) pig iron, crude steel, and rolled steel belong to the sector of *Ferrous Metal Smelting and Processing*; and (9) refined copper, primary aluminum, and alumina oxide belong to the sector of *Nonferrous Metal Smelting and Processing*.

## Data Records

**Fig. 2:**
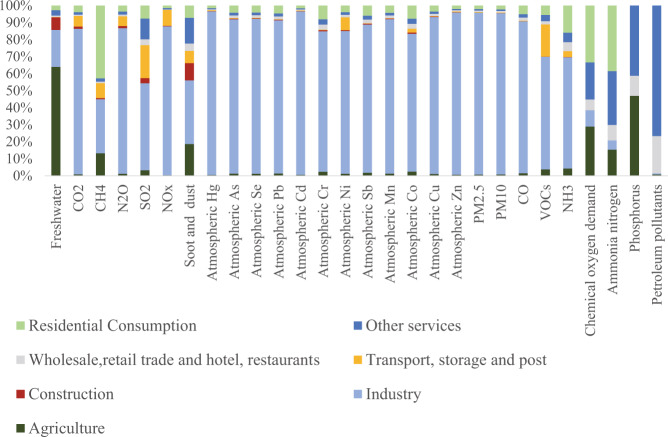
Sectoral share of the 28 environmental pressures in 2017.

The 2017 and 2018 CEEIO databases contain a total of 31072 data covering 44 and 28 environmental pressures in 45 sectors, 49 sectors, 91 sectors, 96 sectors and 149 sectors (153 for 2018), which are stored in files ‘2017 Chinese environmentally extended input-output database’ and ‘2018 Chinese environmentally extended input-output database’ respectively^45^. Figshare file 6^42^ shows the amounts of 38 environmental pressures in 49 sectors and the top three sectors in the 2017 database^42^. Figure 2 shows the share of the sectoral share of the 28 environmental pressures. We find that: (1) more than half (64.0%) of the freshwater consumption comes from the agricultural sector; (2) manufacturing is the major source (85.6%) of air pollutants; and (3) water pollutants mainly (84.3%) come from households, agriculture, and other services.

**Fig. 3:**
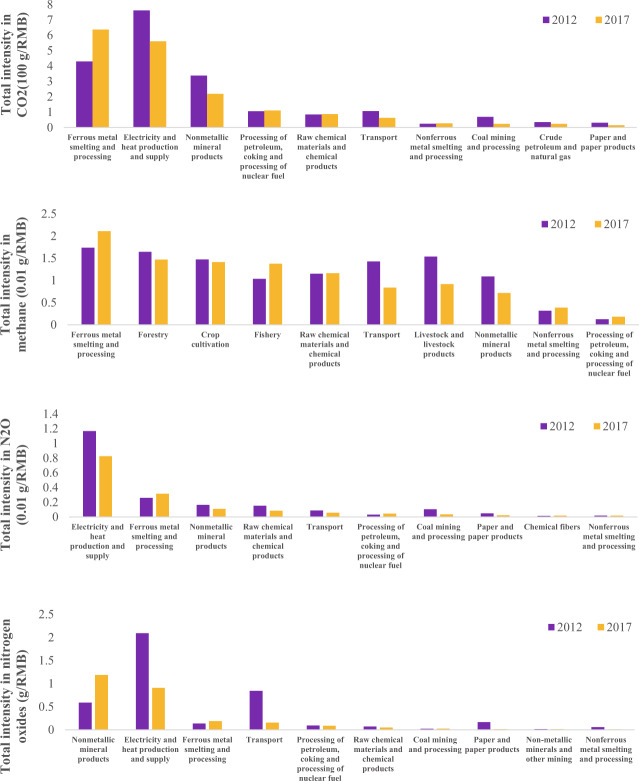
GHG and NO_x_ intensities of top 10 sectors in the 2012 and 2017 CEEIO databases. (**a**) total intensity in CO_2_, (**b**) total intensity in CH_4_, (**c**) total intensity in N_2_O, (**d**) total intensity in NO_x_.

Figure 3 shows the total greenhouse gas (GHG) and NO_x_ intensities of the top 10 sectors in 2012 and 2017. Compared with the 2012 CEEIO database, the GHG and NO_x_ intensities of the *Electricity & Heat Production and Supply* sector drop by 31.9% on average, while those of the *Ferrous Metal Smelting and Processing* sector increase by 31.6% on average.

## Technical Validation

### Uncertainties

There are three major sources of uncertainty in our data.

1. According to the IPCC^23^ and relevant studies^46–48^, there are uncertainties in emission factors of GHGs and HTEs for energy combustion and industrial processes, as well as the amount of industrial products. For instance, Supplementary Table 4 shows the uncertainties in CO_2_ emission factors for fuel combustion in energy industry^16,17^. Table 1 shows the coefficient of variation of the data used to estimate HTEs emissions.

**Table 1:** The coefficient of variation of the data used to estimate HTES emissions.

Categories	Parameter description	coefficient of variation
**Coal combustion sources**		
Coal consumption	power plant	5%
	Industrial sectors	5%
	Residential sectors	14%
	Other sectors	16%
**Non-coal combustion sources**		
Liquid fuel combustion	Liquid fuel consumption	5%
	Emission factors	25%
Nonferrous metal smelting	Nonferrous metal production	5%
Non-metallic minerals manufacturing	Output of Cement/glass/brick	20%
	emission factors (cement, glass)	25%
	emission factors (brick)	30%
Ferrous metal smelting	Pig iron and steel yield	15%
2. The data on national energy consumption published by the NBSC are not consistent with the sum of energy consumption published by 30 province. This means that, due to different statistical caliber and systematic deviation in the statistical process, there are uncertainties in the energy consumption data of each sector.
3. When extending the 49-sector CEEIO database to the 149-sector CEEIO database for 2017 (153 sectors for 2018), and merging the 149-sector CEEIO database into 45-sector, 91-sector, and 96-sector CEEIO database, we assume that the intensities of environmental pressures of the sub-sectors split from the aggregate sectors are the same as those in the aggregate sectors. This treatment cannot take the differences among the sub-sectors into account.

**Fig. 4:**
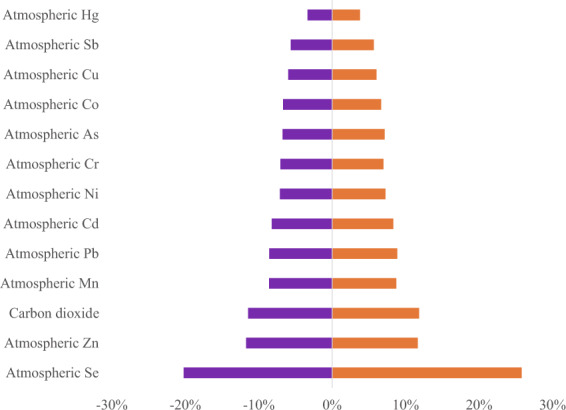
The 97.5% confidence intervals of the emission of 13 environmental pressures.

It is difficult to quantify the uncertainty caused by merging and splitting sectors as there are not enough data to measure the difference between sub-sectors.The Monte Carlo simulation has been applied to quantify the variations of emission inventory due to the uncertainties in emission factors and activity data. All variables are assumed to follow a normal distribution in our analysis. Based on the coefficient of variation collected from relevant research, 100000 random samples of the variables have been generated to estimate the range and distribution of emissions of 13 types of environmental pressures. Figure 4 shows the 97.5% confidence intervals of the emissions of 13 environmental pressures. The uncertainty of Atmospheric Se emissions turns to be the highest (−20.2%~25.8%), while the Atmospheric Hg has a relatively lower uncertainty (−3.4%~3.8%).

### Comparisons with existing emission datasets

**Fig. 5:**
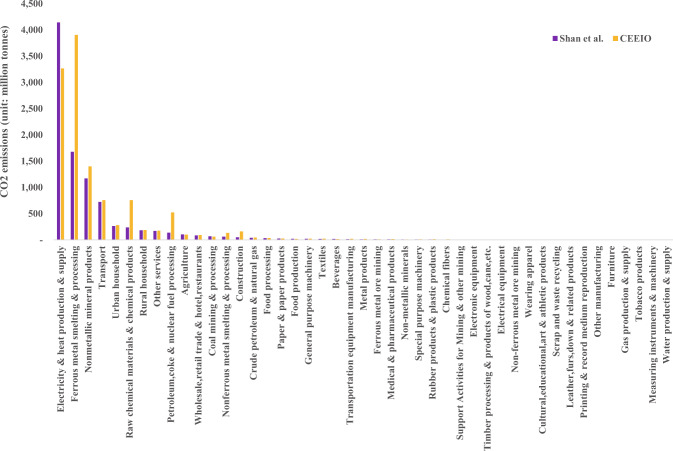
CO_2_ emissions of each sector of China in 2017 in our database.

**Fig. 6:**
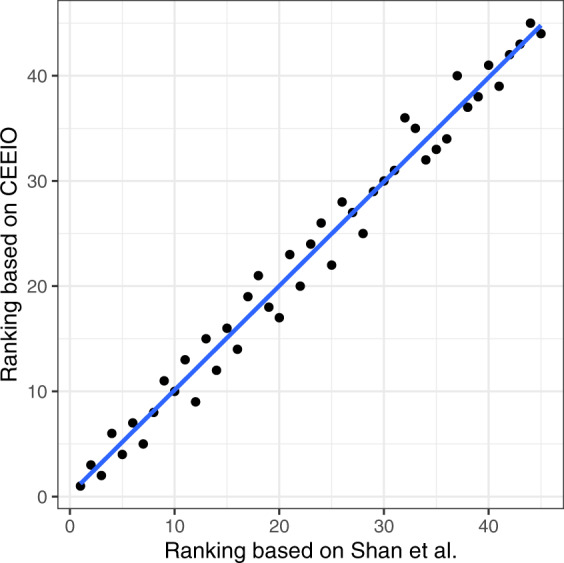
Kendall correlation coefficients of the CO_2_ emissions from each sector between the 2017 CEEIO database and Shan *et al*. (p < 2.2 × 10^−22^).

Figure 5 shows sectoral CO_2_ emissions of China in 2017 in this study and Shan *et al*.^16,17^. The total CO_2_ emissions in China based on this paper are 2.8 billion tons (29.65%) more than those in Shan *et al*. . The difference mainly comes from the fact that, besides cement clinker, we consider CO_2_ emissions from additional industrial processes, such as processes of making lime, glass, ammonia, soda ash, ethylene, coke, pig iron, crude steel, and primary aluminum (2,557 million tons), while Shan *et al*. did not. We also consider emissions from the consumption of liquefied natural gas (156 million tons) while Shan *et al*. did not. Figure 6 shows the Kendall correlation analysis of the CO_2_ emissions in the two databases, which shows that the results of the two databases are highly correlated.

## Limitations and future work

1. The amount of freshwater consumption, SO_2_, soot & dust, industrial waste, and 13 water pollutants generated from each sector are obtained by multiplying the corresponding intensities in 2012 with the total output of each sector in 2017 in constant prices based on 2012 and scaled based on aggregate data in 2017. These data are subject to high uncertainty and will be updated after the publication of the 2018 Annual Report on China’s Environmental Statistics.
2. The IPCC published GHG emission factors for industrial processes of products such as nitric acid, methanol, ferroalloys, raw magnesium, lead, zinc. But the amounts of these industrial products are not currently available. Future updates will seek for additional data to estimate GHG emissions from these processes.
3. Household biomass combustion, waste disposal, and fuel combustion from private vehicles all contribute to environmental pressures. However, the relevant consumption data are not readily available. In the future, we will take these emissions into account when relevant data become available.

## Supplementary information

Supplementary Information

## Data Availability

The MATLAB Code used to merge the 149 sectors into 45 sectors, 49 sectors, 91 sectors, and 96 sectors is shown below for transparency and verifiability. We take merging 149 sectors into 96 sectors as an example. % Copyright 2020 Nanchang University. All rights reserved. % *Read the binary matrix from the excel files* MFA = xlsread(‘2017CEEIO.xlsx’,‘149S-96SMM’,‘B2:ET97’); *% MFA refers to the binary matrix used to merge 149 sectors into 96 sectors* %Read the data in the 2017 Chinese benchmark IO table and environmental database IIA = xlsread(‘2017CEEIO.xlsx’,‘149DIOT’,‘D7:EV155’); *% IIA refers to the intermediate input matrix in the 2017 Chinese benchmark IO table* TIUA = xlsread(‘2017CEEIO.xlsx’,‘149DIOT’,‘EW7:EW155’); *% TIUA refers to the total intermediate uses vector in the 2017 Chinese benchmark IO table* FUA = xlsread(‘2017CEEIO.xlsx’,‘149DIOT’,‘EX7:FG155’); *% FUA refers to the final uses matrix in the 2017 Chinese benchmark IO table* IMA = xlsread(‘2017CEEIO.xlsx’,‘149DIOT’,‘FH7:FH155’); *% IMA refers to the import vector in the 2017 Chinese benchmark IO table* GOA = xlsread(‘2017CEEIO.xlsx’,‘149DIOT’,‘FI7:FI155’); *% GOA refers to the gross output vector in the 2017 Chinese benchmark IO table* TIIA = xlsread(‘2017CEEIO.xlsx’,‘149DIOT’,‘D156:EV156’); *% TIIA refers to the total intermediate input vector in the 2017 Chinese benchmark IO table* VAA = xlsread(‘2017CEEIO.xlsx’,‘149DIOT’,‘D157:EV161’); *% VAA refers to the value added matrix in the 2017 Chinese benchmark IO table* TIA = xlsread(‘2017CEEIO.xlsx’,‘149DIOT’,‘D162:EV162’); *% TIA refers to the total input vector in the 2017 Chinese benchmark IO table* EPA = xlsread(‘2017CEEIO.xlsx’,‘149-S 2017CEEIOT(RMB)’,‘D163:EV206’); *% EPA refers to the environmental pressure matrix in the 2017 Chinese EEIO table of 149 sectors* %Merge the 149 sectors into 96 sectors IIFA = MFA*IIA*(MFA)’; *% IIFA refers to the intermediate input matrix in the 2017 Chinese EEIO table of 96 sectors* TIUFA = MFA*TIUA; *% TIUFA refers to the total intermediate uses vector in the 2017 Chinese EEIO table of 96 sectors* FUFA = MFA*FUA; *% FUFA refers to the final uses matrix in the 2017 Chinese EEIO table of 96 sectors* IMFA = MFA*IMA; *% IMFA refers to the import vector in the 2017 Chinese EEIO table of 96 sectors* GOFA = MFA*GOA; *% GOFA refers to the gross output vector in the 2017 Chinese EEIO table of 96 sectors* TIIFA = TIIA*(MFA)’; *% TIIFA refers to the total intermediate input vector in the 2017 Chinese EEIO table of 96 sectors* VAFA = VAA*(MFA)’; *% VAFA refers to the value added matrix in the 2017 Chinese EEIO table of 96 sectors* TIFA = TIA*(MFA)’; *% TIFA refers to the total input vector in the 2017 Chinese EEIO table of 96 sectors* EPFA = EPA*(MFA)’; *% EPFA refers to the environmental pressure matrix in the 2017 Chinese EEIO table of 96 sectors*
