# Supplementary material for: Chinese environmentally extended input-output database for 2017 and 2018
Source: Sci Data. 2021 Sep 30;8:256. doi: 10.1038/s41597-021-01035-1 (PMC8484342; doi:10.1038/s41597-021-01035-1)
Supplement: Supplementary file 1 — Supplementary Information [file 41597_2021_1035_MOESM1_ESM.docx]

# ****Supplementary Information****

# ****for****

# Chinese environmentally extended input-output database for 2017 and 2018

Xi Tian^1,2,3^, Yiwei Liu^3^, Ming Xu^4,5^*, Sai Liang^6^, Yaobin Liu^1,3^

1. Research Center for Central China Economic and Social Development, Nanchang University, Nanchang 330031, China

2. Jiangxi Ecological Civilization Research Institute, Nanchang University, Nanchang 330031, China

3. School of Economics and Management, Nanchang University, Nanchang 330031, China

4. School for Environment and Sustainability, University of Michigan, Ann Arbor, Michigan 48109-1041, United States

5. Department of Civil and Environmental Engineering, University of Michigan, Ann Arbor, Michigan 48109-2125, United States

6. Key Laboratory for City Cluster Environmental Safety and Green Development of the Ministry of Education, Institute of Environmental and Ecological Engineering, Guangdong University of Technology, Guangzhou, Guangdong 510006, China

corresponding author(s): Ming Xu (mingxu@umich.edu)

****Content****

[Supplementary Table 1. The differences in sector classification between the 139-sector 2012 CEEIO database and 149-sector 2017 CEEIO database. 3](#_Toc21691)

[Supplementary Table 2. The differences in sector classification between the 45-sector 2012 CEEIO database and the 49-sector 2017 CEEIO database. 4](#_Toc13473)

[Supplementary Table 3. The differences in sector classification between the 91-sector 2012 CEEIO database and 96-sector 2017 CEEIO database. 5](#_Toc22371)

[Supplementary Table 4. CO](#_Toc5935)_[2](#_Toc5935)_ [emission factors in fuel combustion. 6](#_Toc5935)

## **Supplementary Table** 1. The differences in sector classification between the 139-sector 2012 CEEIO database and 149-sector 2017 CEEIO database.

| Sectors in the 139-sector 2012 CEEIO database | Sectors in the 149-sector 2017 CEEIO database |
| --- | --- |
| Non-alcoholic beverage | Non-alcoholic beverage |
|  | Tea |
| Cultural products, toys, sporting and athletic and recreational products | Culture and education, sports and recreational products |
| Wholesale and retail trade | Wholesale |
|  | Retail |
| Railway transport | Railway transport for passengers |
|  | Railway transport for cargo and auxiliary activities |
| Highway transport | Urban public transport and highway transport for passengers |
|  | Highway transport for cargo and auxiliary activities |
| Water transport | Water transport for passengers |
|  | Water transport for cargo and auxiliary activities |
| Air transport | Air transport for passengers |
|  | Air transport for cargo and auxiliary activities |
| Handling and transport agent | Multimodal transport and transport agents |
| Warehousing | Handling and warehousing |
| Software and information technology services | Software services |
|  | Information technology services |
|  | Arts and crafts |
|  | Broadcasting, television and satellite transmission services |
|  | The Internet and related services |

## **Supplementary Table** 2. The differences in sector classification between the 45-sector 2012 CEEIO database and the 49-sector 2017 CEEIO database.

| Sectors in the 45-sector 2012 CEEIO database | Sectors in the 49-sector 2017 CEEIO database |
| --- | --- |
| Crop cultivation | Crop cultivation |
| Forestry | Forestry |
| Livestock and livestock products | Livestock and livestock products |
| Fishery | Fishery |
|  | Technical services for agriculture |
| Non-metallic minerals and other mining | Non-metallic minerals mining |
|  | Support activities for mining and other mining |
| Rubber products | Rubber products and Plastic products |
| Plastic products |  |
| Transport equipment | Automobile manufacturing |
|  | Railway, shipbuilding, aerospace and other transport equipment manufacturing |
| Cultural products, toys, sporting and athletic and recreational products | Cultural, educational, art, and athletic products |
| Arts and crafts products and other manufacturing products | Other manufacturing products |
|  | Metal products, machinery and equipment repairs |
| Other services | Wholesale, retail trade and hotel, restaurants |
|  | Other services |

## **Supplementary Table** 3. The differences in sector classification between the 91-sector 2012 CEEIO database and 96-sector 2017 CEEIO database.

| Sectors in the 91-sector 2012 CEEIO database | Sectors in the 96-sector 2017 CEEIO database |
| --- | --- |
| Crop cultivation | Crop cultivation |
| Forestry | Forestry |
| Livestock and livestock products | Livestock and livestock products |
| Fishery | Fishery |
|  | Technical services for agriculture |
| Non-metallic minerals and other mining | Non-metallic minerals mining |
|  | Auxiliary mining services and other mining |
| Cultural products, toys, sporting and athletic and recreational products | Culture and education, arts, sports and recreational products |
| Arts and crafts products and other manufacturing products | Other manufacturing products |
|  | Metal products, machinery and equipment repairs |
| Air transport and other transport | Air transport |
|  | Multimodal transport and transport agent |
| Warehousing | Handling and warehousing |
| Other services | Information transmission, software and information technology services |
|  | Other services |

## **Supplementary Table** 4. CO_2_ emission factors in fuel combustion.

| Fuel | Default value(g CO_2_/Gj) | CV |
| --- | --- | --- |
| Raw Coal | 66,178.26 | 17.5% |
| Cleaned Coal | 66,178.26 | 10.8% |
| Other Washed Coal | 66,178.26 | 26.0% |
| Briquettes | 66,178.26 | 18.3% |
| Crude Oil | 73,300.00 | 1.1% |
| Nature Gas | 63,529.75 | 15.0% |
| Coke | 107,000.00 | 3.4% |
| Other Coking Products | 80,700.00 | 23.1% |
| Gasoline | 69,300.00 | 2.0% |
| Kerosene | 71,900.00 | 1.2% |
| Diesel Oil | 74,100.00 | 1.3% |
| Fuel Oil | 77,400.00 | 2.2% |
| Naphtha | 73,300.00 | 3.9% |
| Lubricants | 73,300.00 | 3.9% |
| Paraffin Waxes | 73,300.00 | 3.9% |
| White spirit | 73,300.00 | 3.9% |
| Bitumen | 80,700.00 | 3.9% |
| Petroleum Coke | 97,500.00 | 3.9% |
| LPG | 63,100.00 | 7.7% |
| Other Petroleum Products | 73,300.00 | 3.9% |
| Coke Oven Gas | 44,400.00 | 33.4% |
| Blast Furnace Gas | 260,000.00 | 33.4% |
| Converter Gas | 260,000.00 | 33.4% |
| Other Gas | 260,000.00 | 33.4% |
| Refinery Gas | 57,600.00 | 5.5% |
